# Response to selection while maximizing genetic variance in small populations

**DOI:** 10.1186/s12711-016-0248-3

**Published:** 2016-09-20

**Authors:** Isabel Cervantes, Juan Pablo Gutiérrez, Theo H.E. Meuwissen

**Affiliations:** 1 Department of Animal Production, Faculty of Veterinary, Complutense University of Madrid, Avda. Puerta de Hierro s/n, 28040 Madrid, Spain; 2 Department of Animal and Aquacultural Sciences, Norwegian University of Life Sciences, Box 1432, Ås, Norway

## Abstract

**Background:**

Rare breeds represent a valuable resource for future market demands. These populations are usually well-adapted, but their low census compromises the genetic diversity and future of these breeds. Since improvement of a breed for commercial traits may also confer higher probabilities of survival for the breed, it is important to achieve good responses to artificial selection. Therefore, efficient genetic management of these populations is essential to ensure that they respond adequately to genetic selection in possible future artificial selection scenarios. Scenarios that maximize the maximum genetic variance in a unique population could be a valuable option. The aim of this work was to study the effect of the maximization of genetic variance to increase selection response and improve the capacity of a population to adapt to a new environment/production system.

**Results:**

We simulated a random scenario (A), a full-sib scenario (B), a scenario applying the maximum variance total (MVT) method (C), a MVT scenario with a restriction on increases in average inbreeding (D), a MVT scenario with a restriction on average individual increases in inbreeding (E), and a minimum coancestry scenario (F). Twenty replicates of each scenario were simulated for 100 generations, followed by 10 generations of selection. Effective population size was used to monitor the outcomes of these scenarios. Although the best response to selection was achieved in scenarios B and C, they were discarded because they are unpractical. Scenario A was also discarded because of its low response to selection. Scenario D yielded less response to selection and a smaller effective population size than scenario E, for which response to selection was higher during early generations because of the moderately structured population. In scenario F, response to selection was slightly higher than in Scenario E in the last generations.

**Conclusions:**

Application of MVT with a restriction on individual increases in inbreeding resulted in the largest response to selection during early generations, but if inbreeding depression is a concern, a minimum coancestry scenario is then a valuable alternative, in particular for a long-term response to selection.

## Background

Preserving genetic diversity in a population is one of the main objectives for a breed conservation program. It guarantees availability of genetic variation in case the population needs to adapt to a new environment/production system, thus increasing its survival. The existence of large amounts of genetic variation in animal genomes is the basis for the survival and development of animal populations as well as for the versatility of livestock species, including adaptation to varying environments via natural selection. In general, response to artificial selection on a trait is directly proportional to the genetic standard deviation of the trait [1].

Developing and implementing mating criteria that pair selected parents to maintain/increase genetic variance are worthwhile because mating parents appropriately can further improve genetic gain and/or reduce inbreeding [2, 3]. Methods to maximize genetic variance have been used in simulated metapopulations [4] or in a unique population [5], but their usefulness in terms of response to selection needs to be tested. Livestock populations are often involved in artificial selection, with the purpose of improving the performance of individuals for one or more traits. Even in populations under conservation programs, where the objective is to maximize genetic variability, animals that are far from the standard of the breed are discarded. Moreover, breeders are interested in selecting for traits that are related to fitness or in maintaining acceptable levels of performance for productive traits [6]. In these scenarios, under artificial selection conditions, methods to maximize the genetic variance would perform differently. Management of small populations also focuses on reducing the impact of inbreeding depression (resulting from high individual inbreeding coefficients), i.e. limiting increases in inbreeding across generations. Thus, monitoring the effective population size has become one of the most important issues in the management of small populations. In particular, it is a measure of the long-term performance of a population in terms of both diversity and inbreeding. It can be used as a way to characterize the risk status of livestock breeds [1, 7, 8] and analyze the genetic health of populations. However, there are very few studies that have monitored genetic variability in populations under selection.

The objective of this study was to analyse the impact of different mating policies on genetic variability in small populations. These policies, some of which were novel, can result in greater response under a selection scenario. Our aim was to investigate the effect of the maximization of genetic variance on response to selection by simulating a selection scenario.

## Methods

### Data simulation

A simulation study was performed using a founder population that comprised equal numbers of unrelated individuals of both sexes. Genetic values and phenotypes for a hypothetical additive trait were simulated using heritabilities of 0.10, 0.25 and 0.50. The phenotypic variance was set at 100. Genetic values in the founder population were obtained by the product of the genetic standard deviation (*σ*_*a*_) and ${\text{z}}_{i}$, a random standard normal value ($u_{i} = {\text{z}}_{i} \sigma_{a}$). Genetic values for animals with known parents were computed as:

$$u_{i} = 1/2u_{j} + 1/2u_{k} + \varphi_{i} ,$$

where *j* and *k*, are the parents of *i*, and *u*_*i*_, *u*_*j*_ and *u*_*k*_ are the additive genetic values for individuals *i*, *j* and *k*, and *φ*_*i*_ is the within-family deviation produced by the Mendelian sampling effect in the *i*th individual. *φ*_*i*_ was obtained by the product of the Mendelian standard deviation (*σ*_*φ*_) and ${\text{z}}_{i}$, a random standard normal deviate ($\varphi_{i} = {\text{z}}_{i} \sigma_{\varPhi }$). The Mendelian sampling variance was computed as:

$$var\left( \varphi \right) = 1/2\left( {1 - 1/2\left( {F_{j} + F_{k} } \right)} \right)\sigma_{u}^{2} ,$$

where *F*_*j*_ and *F*_*k*_ are the inbreeding coefficients of the parents of individual *i*.

The phenotype was computed as $y_{i} = 100 + u_{i} + {\text{z}}_{i} \sigma_{e}$, where the mean value was 100, *u*_*i*_ is the genetic value, $Z_{i}$ is a random standard normal value, and *σ*_*e*_ is the residual standard deviation.

### Mate selection scenarios

After 100 discrete generations without selection and with 50 males and 50 females born each generation, we performed a mate selection approach, in which mating and defining the number of offspring produced were done in one step by taking the optimization objective for one of the following six scenarios into account [6, 9]:

- *Scenario A* a random mating scenario in which a sire and a dam were sampled randomly for each offspring, with no limits to the number of animals that can act as parents and to the size of the family.
- *Scenario B* a full-sib family scenario in which 50 full-sib lines with two full-sibs per family (one of each sex) were produced by mating the full-sibs each generation.
- *Scenario C* applied the maximum variance total (MVT) method that was developed by Bennewitz and Meuwissen [4] and modified by Cervantes and Meuwissen [5] to maximize the genetic variance in the population. The contribution of parents was optimized by maximizing the genetic variance criterion by optimizing the number of offspring from each possible mating pair. There was neither a minimum nor a maximum number of parents and no limitation on family size. To predict the additive genetic variance in the next generation, we used the n x n (n = 100) coancestry matrix among the offspring (not among the current parents), ${\mathbf{M}}$. The off-diagonal elements of ${\mathbf{M}}$ are the coancestries between offspring and are equal to the inbreeding coefficients of the hypothetical offspring of each mating pair. Diagonal elements of ${\mathbf{M}}$ are the self-coancestries of each offspring (*M*_*ii*_), the inbreeding coefficients of self-mated individuals; they also reflect the individual inbreeding coefficients (*F*_*i*_) ($F_{i} = 2{\text{M}}_{ii} - 1$) [5]. Therefore, in this scenario, at each generation, the following expression was maximized by optimizing the contribution of parents to the next generation using an annealing algorithm [5]:
  $${\text{var}}\left( {u_{W} } \right) = \frac{1}{n}\mathop \sum \limits_{i = 1}^{n} \left[ {\left( {1 + F_{i} } \right) - 2\overline{M} } \right],$$
  where ${\text{var}}\left( {u_{W} } \right)$ is the genetic variance for a hypothetical quantitative trait in the offspring generation, *F*_*i*_ is the inbreeding coefficient of individual *i*, $\overline{M}$ is the average of all elements of the coancestry matrix ${\mathbf{M}}$, and *n* is the number of individuals produced per generation (*n* = 100).
- *Scenario D* applied the MVT method with restriction on the increase in average inbreeding per generation by maximizing the following expression:
  $$\begin{aligned} {\text{C}}\_{\text{MVT}} + \Delta F& = \frac{1}{n}\mathop \sum \limits_{i = 1}^{n} \left[ {\left( {1 + F_{i} } \right) - 2\overline{M} } \right] \\ & - \lambda \left( {\Delta F_{\text{r}} - \Delta F_{\text{d}} } \right)*I\left( {\Delta F_{\text{r}} > \Delta F_{\text{d}} } \right), \\ \end{aligned}$$
  where $\Delta F_{\text{r}}$, is the increase in inbreeding achieved in the solution, $\Delta F_{\text{d}}$ is the maximum desired rate of inbreeding (0.01), and *I*() denotes an indicator variable that is equal to 1 if the expression between the brackets is true and 0 otherwise. *λ* is the penalization factor and was set equal to 1 (this value was obtained empirically by trial and error until the restriction $\Delta F_{\text{d}} = 0.01$ was satisfied). The increase in inbreeding was computed as:
  $$\Delta F = \frac{{F_{g} - F_{g - 1} }}{{1 - F_{g - 1} }},$$
  where *F*_*g*_ and *F*_*g*−1_ are the average inbreeding coefficients in generations *g* and *g* − 1.
- Scenario E is the same scenario as D but with a restriction on the average individual increase in inbreeding per generation. This parameter standardizes the individual inbreeding coefficient in the individual increase in inbreeding per generation [10, 11]. It is useful in the case of unbalanced pedigree depth [12], but here it avoids wrong deductions due to averaging too extreme probability values as are inbreeding coefficients. Here, *λ* = 60 was used in the objective function of scenario D, which ensures that the restriction $\Delta F_{i} *_{d} = 0.01$ is achieved. This *λ* was much larger than above because of the much lower scale of the individual increase in inbreeding. *ΔF* *  is the average of the individual increase in inbreeding computed as:
  $$\Delta F_{i} = 1 - \sqrt[{g_{i} - 1}]{{1 - F_{i} }},$$
  where *g*_*i*_ is the number of generations in the pedigree of individual *i* and *F*_*i*_ the inbreeding coefficient of individual *i*.
- Scenario F is a minimum coancestry selection scenario, where the objective function was to minimize the increase in inbreeding between generations by minimizing the mean coancestry between the offspring in the next generation, including self-coancestry ($\overline{M}$). No restriction was made on the number of animals that can act as parents or on family size.

In scenarios C, D and E, the mating procedure maximized the genetic variance, but with restrictions on inbreeding for scenarios D and E. Objective function maximization was performed by the annealing algorithm [13, 14], which optimized the mating design for each generation. The mechanics of the annealing algorithm begins with a random initial solution and with a small random change that designs an alternative solution. This alternative solution is compared with the initial solution and accepted if it is better. However, to avoid local maxima in the objective function, it also accepts solutions that are worse with a probability equal to $\varOmega = e^{{\frac{ - \Delta }{\text{T}}}}$, where $\Delta$ is the difference in value of the objective function between the alternative solution and the initial solution and ${\text{T}}$ is the temperature. The simulated annealing algorithm was implemented using an initial temperature of 0.001 and was reduced by a factor 0.01 in each step. Blocks of 1000 iterations were run. Many different solutions share the optimum solution and the algorithm performs a random solution within them. This annealing algorithm was used for all scenarios that involved optimization (Scenarios C, D, E and F), even in Scenario F in which the objective function only involved minimizing the increase in inbreeding between generations. Some additional runs were carried out with 10,000 iterations for Scenarios D and E in order to obtain more refined solutions to draw the histograms of the distribution of inbreeding coefficients.

### Selection

Twenty replicates of each mating scenario described above were simulated for 100 generations, followed by an additional 10 generations of selection. From generation 101 onwards, 20 % of the individuals with the highest phenotypic value were kept for breeding and randomly mated producing 50 males and 50 females for the next generation. The 20 replicates of each scenario were used to measure the uncertainty in the response to selection for comparisons between scenarios.

Response to selection was assessed from the average breeding value per generation. The average breeding value in each generation was set equal to zero such that all the scenarios started at the same origin. Results were analyzed using the STATISTICA v. 8.0 package [15].

### Effective population size

In order to monitor the remaining genetic diversity, the effective population size was computed for each generation for the simulated scenarios, using both the individual increase in inbreeding [10–12] and the increase in pairwise coancestry [16]. The individual increase in inbreeding is defined as $\Delta F_{i} = 1 - \sqrt[{g_{i} - 1}]{{1 - F_{i} }}$, where *g*_*i*_ is the number of generations in the pedigree of individual *i* since the founder individuals, and *F*_*i*_ is the inbreeding coefficient of individual *i* [11]. The increase in coancestry between any pair of individuals *j* and *k* was computed as:

$$\Delta c_{jk} = 1 - \sqrt[{\left( {\frac{{g_{j} + g_{k} }}{2}} \right)}]{{1 - c_{jk} }},$$

where *c*_*jk*_ is the inbreeding coefficient of a hypothetical offspring from the mating of individuals *j* and *k*, and *g*_*j*_ and *g*_*k*_ are the generation number of each parent (in this case, we generate the same discrete generation for both) [16]. Both parameters excluded self-fertilization. Using the average of the individual increase in inbreeding and the average increase in pairwise coancestry for all pairs of individuals in a reference subpopulation (last generation before and after selection), effective population size based on individual increases in inbreeding (realized effective size) and on increases in coancestry were computed as $\overline{{N_{e} }} = \frac{1}{{2\Delta \overline{F} }}$ and $\overline{{N_{ec} }} = \frac{1}{2\Delta \overline{c} },$ respectively [10–12, 16].

The method that we applied only focused on restricting inbreeding. There were no explicit restrictions on family size but the method creates an equal family size, therefore, doubling the effective population size in Scenario F. Allowing for variance in family size would have reduced effective size by definition.

Genetic variance, effective population size (both based on individual increases in inbreeding and in pairwise coancestry) and genetic response were analyzed to compare the scenarios. Effective population size analyses were performed using the ENDOG program (version v4.8) [17, 18]. An R program was used to draw the distribution of inbreeding coefficients [19].

## Results

### Family size

Table 1 shows the family structure and the percentages of inbred matings for each simulated scenario before selection. The average number of offspring per family was 2 in all scenarios except in Scenarios A and C, where the average numbers of offspring per family were equal to 2.3 and 2.2, respectively. The number of individuals acting as parents ranged from 86 % for Scenario A to 99 % for Scenario F. Regarding family size in Scenario C, 54 and 35 % of the matings had two and three offspring, respectively. In Scenario D, in which a restriction of 0.01 was applied to the rate of inbreeding, 81 % of the matings had two offspring, while in Scenario E it was 67, this scenario considering the average individual increase in inbreeding per generation to perform the restriction. With Scenario F, it was expected that all individuals would contribute two descendants, but the algorithm did not cover 100 % of the population. In return, this slightly lower performance made it possible for the algorithm to explore the whole parameter space.

**Table 1:** Family structure and percentage of inbred matings in the six simulated scenarios

Scenario	A	B	C	D	E	F
Average number of offspring	2.3	2.0	2.2	2.0	2.0	2.0
Individuals acting as parents	86 %	100 %	88 %	98 %	97 %	99 %
Individuals having two offspring	32 %	100 %	54 %	81 %	67 %	99 %
Inbred matings	0.1 % full-sibs and 4.8 % half-sibs	100 % full-sibs	85 % full-sibs and 13 % half-sibs	2.5 % full-sibs and 11.0 % half-sibs	2.7 % full-sibs and 12.9 % half-sibs	2.4 % half- sibs

*A* random scenario, *B* full-sib scenario, *C* maximum variance total scenario, *D* maximum variance total limiting the increase in average inbreeding scenario, *E* maximum variance total limiting the average individual increase in inbreeding scenario, *F* minimum coancestry scenario

Finally the proportion of inbred matings was higher in Scenario C (85 % of the matings were between full-sibs and 13 % between half-sibs) than in Scenarios D (2.5 % between full-sibs and 11 % between half-sibs) or E (2.7 % between full-sibs and 12.9 % between half-sibs). The smallest percentage was obtained in Scenario F (only 2.4 % of the matings were between half-sibs). Results for Scenario B (full-sibs) are obvious and not commented here.

### Genetic variance and population structure

**Fig. 1:**
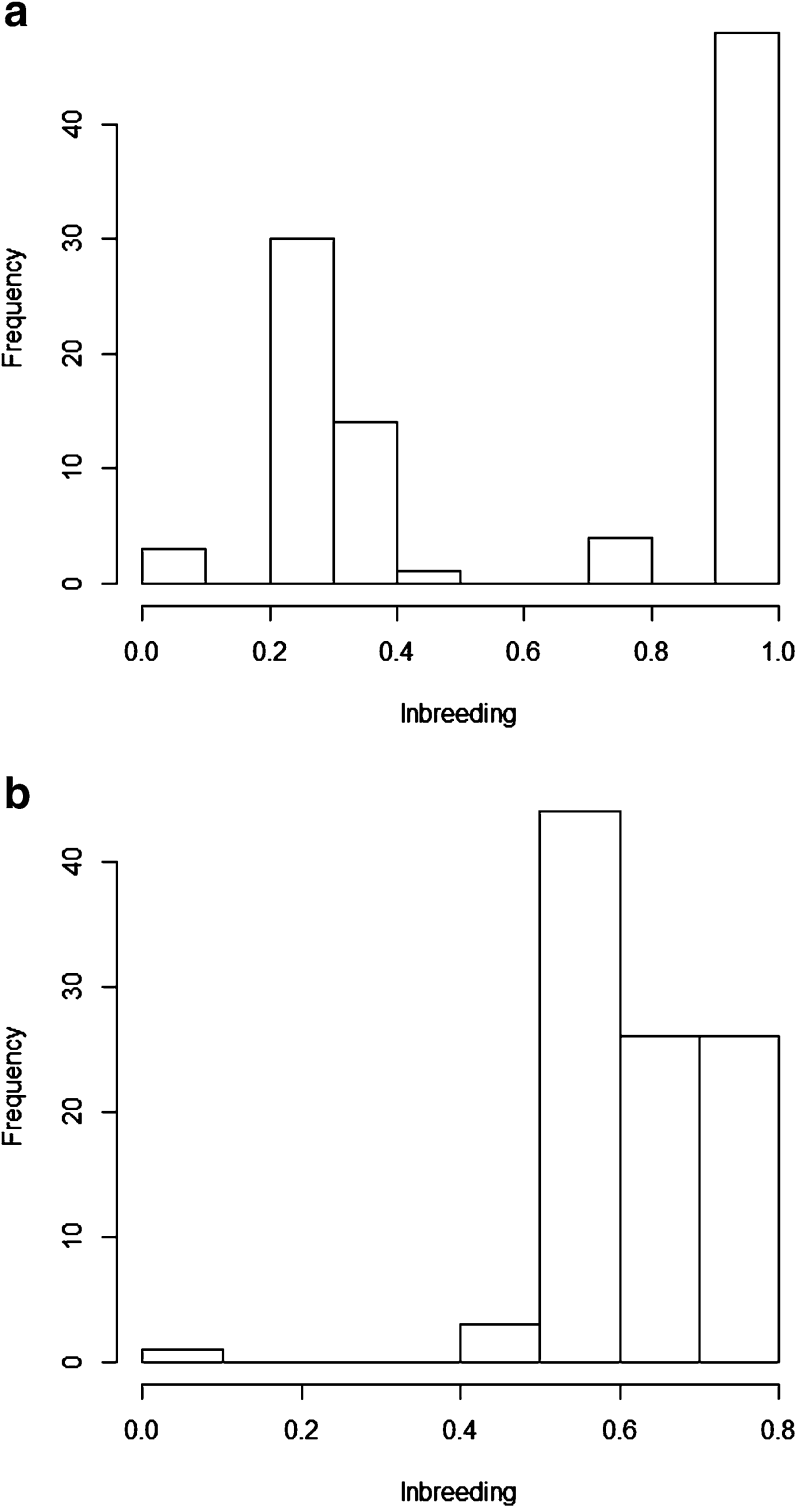
Distribution of inbreeding coefficients in the last generation before selection in Scenarios D (**a**) and E (**b**). Scenario D: with restriction on increase in average inbreeding Scenario E: with restriction on the average individual increase in inbreeding

Table 2 shows the genetic variance averaged across 20 replicates and the average effective population sizes in the last generation for each scenario before selection started and after 10 generations of selection, for a heritability of 0.25. Genetic variance was higher in scenarios that maximized the genetic variance (Scenarios C, D and E) than in Scenarios A and F that did not (random or minimum coancestry). Maximum genetic variances were reached with Scenarios B and C without a restriction on inbreeding. Similar genetic variances were found in Scenarios D and E with a restriction on the mean increase in inbreeding or the mean individual increase in inbreeding, respectively. Effective population sizes based on inbreeding ($$N_{e} )$$ ranged from 2.6 to 193.2 and effective population sizes based on coancestry (*N*_*ec*_) ranged from 100.5 to 258.6 for the last generation before selection. Note that random and minimum coancestry scenarios (A and F) were not structured, whereas Scenarios B and C presented a high level of subdivision. The full-sibs scenario (Scenario B) was completely structured and when the genetic variance was maximized without restriction (Scenario C) almost full-sibs families were obtained. Scenarios D and E involved preferential mating within families and a certain level of subdivision was observed. For the minimum coancestry scenario (Scenario F), the effective population size was almost double the number of individuals (around 200) before selection because 99 % of the matings resulted in two descendants each. After selection, in Scenarios B, C, D and E, all *N*_*e*_ were larger than before selection started. This is because, before selection, the population structure was forced by the method of maximization of genetic variance, and it was modified during selection (random mating between selected animals). Also, after selection, *N*_*ec*_ were similar to those computed based on inbreeding, which indicates that randomly mating selected individuals causes the population structure to disappear. Effective population sizes based on individual increases in inbreeding were larger after than before selection, except in Scenarios A and F. The extreme values of *N*_*e*_ and *N*_*ec*_ (around 400 and 300, respectively) observed in Scenarios B and C in the last generation after selection and using lower intensities (results not shown) can be explained because they reached an inbreeding coefficient of 1 (full-sib families) before selection and a very low inbreeding coefficient after selection (the subdivision disappears with random mating during selection). The realized *N*_*e*_, differed between Scenarios D and E because of the irregular bimodal distribution of individual inbreeding coefficients for Scenario D, with many individuals having extreme inbreeding coefficients and some much lower inbreeding coefficients. Half of the individuals were highly inbred (level of inbreeding greater than 80 %) and the other half were less inbred, between 20 and 40 % (Fig. 1a). A different distribution was observed for Scenario E (Fig. 1b), for which all inbreeding coefficients ranged from 40 to 90 %, with an approximately unimodal distribution.

**Table 2:** Average genetic variance and average effective population sizes (based on individual increases in inbreeding, *N*_*e*_, and increases in coancestry, *N*_*ec*_) in the last generation before and after selection of the best 20 % of individuals for six scenarios and a heritability of 0.25

Scenario	A	B	C	D	E	F
*Before selection*						
Genetic variance	0.60 ± 0.00	1.96 ± 0.00	1.92 ± 0.00	1.24 ± 0.01	1.22 ± 0.01	0.76 ± 0.00
*N*_*e*_	100.33 ± 1.27	2.61 ± 0.00	3.19 ± 0.13	24.29 ± 20.82	50.00 ± 0.00	193.15 ± 2.30
*N*_*ec*_	100.49 ± 0.88	258.60 ± 0.00	122.28 ± 3.86	111.76 ± 13.72	187.79 ± 1.85	194.26 ± 0.18
*After selection*						
Genetic variance	0.44 ± 0.01	0.67 ± 0.04	0.63 ± 0.05	0.50 ± 0.09	0.50 ± 0.08	0.56 ± 0.02
*N*_*e*_	71.46 ± 3.03	159.82 ± 28.68	140.21 ± 26.26	91.30 ± 21.58	89.07 ± 20.98	105.83 ± 5.26
*N*_*ec*_	70.12 ± 2.69	151.33 ± 23.85	133.66 ± 24.43	88.62 ± 20.11	86.20 ± 19.24	102.66 ± 5.00

*A* random scenario, *B* full-sib scenario, *C* maximum variance total scenario, *D* maximum variance total limiting the increase in average inbreeding scenario, *E* maximum variance total limiting the average individual increase in inbreeding scenario, *F* minimum coancestry scenario

### Genetic response

**Fig. 2:**
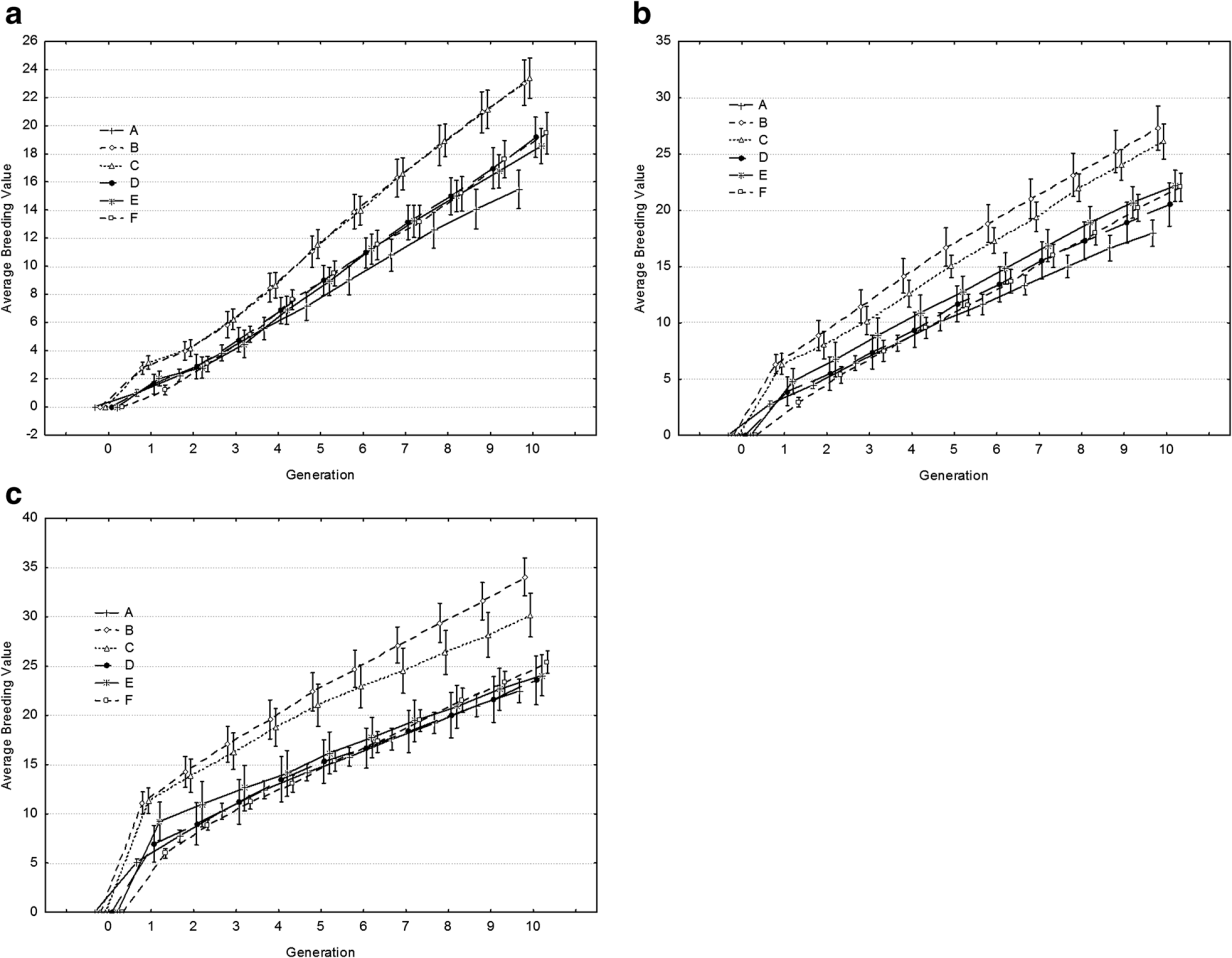
Average breeding value per generation when the 20 % best individuals are kept for breeding. **a** heritability = 0.10; **b** 0.25; **c** 0.50. *A* random scenario, *B* full-sib scenario, *C* maximum variance total scenario, *D* maximum variance total limiting the increase in average inbreeding scenario, *E* maximum variance total limiting the average individual increase in inbreeding scenario, *F* minimum coancestry scenario

Figure 2 shows the average breeding value per generation with the 20 % best individuals kept for breeding. In all cases, the best genetic response was obtained in Scenarios B and C, although these two scenarios cannot be implemented in practice given the very high levels of inbreeding. The random scenario had the lowest genetic response. For a heritability of 0.10, no differences were found between Scenarios D, E and F (Fig. 2a). For heritabilities of 0.25 (Fig. 2b) and 0.50 (Fig. 2c), Scenario E yielded the third best genetic response after Scenarios B and C, although responses for these three scenarios were not significantly different from each other. Scenario E yielded a higher genetic response than Scenarios D and F during the early generations of selection (Fig. 2b, c). Scenario F had a slightly higher genetic response in the last generations (Fig. 2c).

## Discussion

Previous studies evaluated the expected genetic gain under random, compensatory and minimum coancestry mating [2, 3, 20] and concluded that non-random mating decreased the rate of inbreeding but had little effect on genetic response to selection on best linear unbiased predictions (BLUP) and on phenotype [2]. Putting a constraint on selection was not expected to reduce the rate of inbreeding, but the improved family structure due to non-random mating was expected to improve genetic response [3]. The triggering event leading a population to be selected after being preserved could occur in an unknown long future. Thus, there is no limit in the hypothetical number of generations preserving the genetic variance. Therefore, it would be useful to know which management scheme would be the most efficient in terms of selection response. In the current study, we show which scenario would respond best to an environmental change or a selection process, after a long period of management. This is the first time that scenarios of maximization of genetic variance are evaluated in terms of future genetic response and proposed as mating strategies in small populations.

Our results show that the highest genetic response was achieved in Scenarios B and C because they retained more genetic variance. This is well known for scenarios with full-sib lines [21] and was shown for the MVT method in a previous study [5]. However, the $N_{e}$ was drastically reduced and the level of inbreeding approached 100 % as a consequence of the mating designs. Inbreeding reduces the within-population genetic variance by making the population more homozygous. Thus, because the number of deleterious alleles increases and inbreeding depression occurs with these scenarios [22], they are not viable and are only interesting from a theoretical point of view. The MVT method is not suggested as a possible management system for populations under conservation because of the increase in inbreeding (Scenario C). In this study, we also tested two scenarios, D and E, with a restriction on inbreeding. Bennewitz and Meuwissen [4] showed that MVT and the Eding et al. [23] core set method were similar to some extent, but the MVT method prioritizes the conservation of breeds that show large differences in the population mean of the simulated quantitative trait. This maximizes the selection response for a breeding objective, which could make the MVT method attractive, provided inbreeding is controlled.

For practical purposes, only Scenarios A, D, E and F can be realistically considered. For Scenario A, selection responses were much lower and effective population size was also often smaller than for Scenarios D, E, and F. Scenario E (with restriction on the average individual increase in inbreeding) showed a better response during the early generations of selection at heritabilities of 0.25 and 0.50. This did not occur in the scenario with the lowest heritability (0.10) because of the slight effect of the selection. Scenario F was slightly better than Scenario E during the last generations although the genetic variance was not maximized prior to selection under this scenario. Scenarios D and E could be recommended for restricting the increase in inbreeding to ensure that an acceptable effective population size is maintained. However, Scenario D yielded a smaller $N_{e}$ before selection although it was designed to maintain a value of 50. This was due to the restriction on the increase in average inbreeding instead of averaging individual increase in inbreeding. Scenario F maintained larger *N*_*e*_ than Scenario E but the genetic variance did not increase compared with Scenarios D and E and did not result in a high genetic response to selection. Scenario E directly benefited from the subdivision produced before selection, but in scenarios where inbreeding depression is present, Scenario F might be preferable because high levels of inbreeding occur later.

The method of choice to compute *N*_*e*_ is the realized effective population size [24]. In situations where it is necessary to limit the inbreeding if inbreeding increases substantially between generations, a restriction can be imposed on the rate of inbreeding and the effective population size can be optimized. However, it has been shown that two populations (same species or breeds) with the same inbreeding level can have completely different levels of inbreeding depression for the same trait [25, 26]; also by adding detrimental effects in simulations (e.g. deleterious alleles), different inbreeding depression levels among populations might be achieved [27]. As shown in Fig. 1a, when the restriction was imposed on the effective population size that was computed from increases in the average inbreeding coefficient between consecutive generations (Scenario D), the annealing algorithm split the population into two groups to meet the imposed restriction, one that included completely inbred animals and one with less inbred animals, but this resulted in a population with a greater increase in inbreeding than desired. However, when the restriction was imposed on the *N*_*e*_ that was derived from the average individual increases in inbreeding, the distribution of inbreeding coefficients became roughly unimodal (Fig. 1b). Therefore, in Scenario D when the effective population size was computed as the realised effective population size, *N*_*e*_ was roughly half the desired size. This shows that assertions made from averages of inbreeding coefficients can be misleading because high inbreeding coefficients have a too big weight on the average value.

Leroy et al. [24], in agreement with Cervantes et al. [16], suggested that, considering the precision of the estimation of effective population size, coancestry-based methods for computing effective population size are preferable to those based on the increase in inbreeding. In particular when there was population substructure, coancestry-based methods for computing effective size clearly resulted in higher values than those based on inbreeding [16]. The method based on coancestry was found to be the most appropriate to compute effective population size when some population substructure is present and if the goal is to remove this substructure. However they require more computer time because computation of coancestry is carried out on many more coefficients than computation of inbreeding. Otherwise, the most useful estimator of the population inbreeding state would be the effective population size based on individual increase in inbreeding, because inbreeding leads to inbreeding depression. The concept of effective population size is usually defined under a regular system and can be used for predictive purposes, e.g. of the risk status of a population. Note that when there is population substructure, a sudden extinction of subpopulations would make a further decrease of the average IBD impossible and therefore the effective population size values based on the individual increase in inbreeding can appropriately quantify the risk of a population in which gene exchange between subpopulations is not possible [28].

Minimum coancestry mating is frequently used to manage small populations [3, 20, 29] and generates at least as much genetic gain as random mating of selected individuals, presumably because it generates less inbreeding [20, 29]. Less inbreeding implies higher levels of additive genetic variation under the additive genetic model, thus increasing the potential to generate genetic gain [30]. This counterbalances any short-term loss in genetic variation that may result from a negative correlation between Mendelian sampling terms of parents that are paired together by minimum coancestry mating [2]. Therefore, loss of genetic gain will not occur by developing mating criteria that reduce inbreeding. Moreover, in the minimum coancestry selection scenario (F), the total genetic variance decreases as ${\text{MVT}} = 1 + {\text{F}} - 2{\text{M}} = 1 - {\text{M}}$, with a maximum of 1 in the founder generation and decreases asymptotically to 0 [5]. It must be pointed out that this procedure avoids a decline in fitness because inbreeding is reduced but it also prevents purging deleterious recessive alleles as they will be less exposed to natural selection [31]. By restricting the individual increase in inbreeding, genetic gain could increase because genetic variance is maximized. Maximizing the genetic variance and restricting the increase in inbreeding, allows achieving an intermediate solution that combines a certain degree of subdivision in the population and an acceptable rate of inbreeding. This ensures more genetic variation, which could be useful in case the population needs to adapt to a new environment or production system i.e. to meet market demands.

## Conclusions

Full-sibs and the MVT method scenarios were discarded because they are not practical, as well as the random scenario because of its low response to selection. The MVT method scenario with restriction on the increase in average inbreeding per generation yielded lower genetic response and a smaller *N*_*e*_ than the MVT method scenario with restriction on the average individual increase in inbreeding, and therefore should also be discarded. The scenario that uses the individual increase in inbreeding in the restriction yielded greater genetic response than the minimum coancestry selection scenario during the early generations, whereas the minimum coancestry scenario benefitted from a better selection response in the last generations of selection. The greater genetic response obtained in the scenario using the individual increase in inbreeding was due to the moderate substructure that was created in the population by the method, i.e. less subdivision than in the scenario with restriction on the increase in average inbreeding. This subdivision tended to disappear when selection started and the criteria for maximizing genetic variance were not applied. We conclude that the best scenario in terms of genetic response is the scenario based on the MVT method with the restriction on the individual increase in inbreeding, but if inbreeding depression, which can be specific to a population or trait, is of a high concern, the minimum coancestry selection scenario would be more appropriate.
